# Antimicrobial and Antioxidant Activities of Endophytic Fungi Associated with *Arrabidaea chica* (Bignoniaceae)

**DOI:** 10.3390/jof9080864

**Published:** 2023-08-21

**Authors:** Raiana Silveira Gurgel, Dorothy Ívila de Melo Pereira, Ana Vyktória França Garcia, Anne Terezinha Fernandes de Souza, Thaysa Mendes da Silva, Cleudiane Pereira de Andrade, Weison Lima da Silva, Cecilia Veronica Nunez, Cleiton Fantin, Rudi Emerson de Lima Procópio, Patrícia Melchionna Albuquerque

**Affiliations:** 1 Programa Graduate Program in Biodiversity and Biotechnology of the Bionorte Network, School of Health Sciences, Amazonas State University, Manaus 69050-010, Brazil; raianagurgel@hotmail.com (R.S.G.); dorothyivila@gmail.com (D.Í.d.M.P.); cleudiane.andrade@hotmail.com (C.P.d.A.); 2 Research Group on Chemistry Applied to Technology, School of Technology, Amazonas State University, Manaus 69050-020, Brazil; avfg.geq19@uea.edu.br (A.V.F.G.); anne.fernandes13@gmail.com (A.T.F.d.S.); tms.geq18@uea.edu.br (T.M.d.S.); rprocopio@uea.edu.br (R.E.d.L.P.); 3 Graduate Program in Biotechnology and Natural Resources of the Amazon, School of Health Sciences, Amazonas State University, Manaus 69050-010, Brazil; cvnunez@gmail.com (C.V.N.); cfantin@uea.edu.br (C.F.); 4 Bioprospection and Biotechnology Laboratory, National Institute of Amazonian Research, Manaus 69067-375, Brazil; weisilva3@gmail.com; 5 Multicentric Graduate Program in Biochemistry and Molecular Biology, School of Health Sciences, Amazonas State University, Manaus 69050-010, Brazil

**Keywords:** endophytes, Amazonian host, phenolic compounds, fungal metabolites, bioprospecting

## Abstract

The endophytic fungal community of the Amazonian medicinal plant *Arrabidaea chica* (Bignoniaceae) was evaluated based on the hypothesis that microbial communities associated with plant species in the Amazon region may produce metabolites with interesting bioactive properties. Therefore, the antimicrobial and antioxidant activities of the fungal extracts were investigated. A total of 107 endophytic fungi were grown in liquid medium and the metabolites were extracted with ethyl acetate. In the screening of fungal extracts for antimicrobial activity, the fungus identified as *Botryosphaeria mamane* CF2-13 was the most promising, with activity against *E. coli*, *S. epidermidis*, *P. mirabilis*, *B. subtilis*, *S. marcescens*, *K. pneumoniae*, *S. enterica*, *A. brasiliensis*, *C. albicans*, *C. tropicalis* and, especially, against *S. aureus* and *C. parapsilosis* (MIC = 0.312 mg/mL). Screening for antioxidant potential using the DPPH elimination assay showed that the *Colletotrichum* sp. CG1-7 endophyte extract exhibited potential activity with an EC_50_ of 11 µg/mL, which is equivalent to quercetin (8 µg/mL). The FRAP method confirmed the antioxidant potential of the fungal extracts. The presence of phenolic compounds and flavonoids in the active extracts was confirmed using TLC. These results indicate that two of the fungi isolated from *A. chica* exhibit significant antimicrobial and antioxidant potential.

## 1. Introduction

Popularly known in Brazil as crajiru, pariri and carajuru, among other names, *Arrabidaea chica* (Bonpl.) B. Verl. (1868) is a native species of the Amazon region that belongs to the Bignoniaceae family. It is characterized as a climbing shrub, and can reach 2 m in height [1]. Its astringent, emollient properties, and its red pigment, which is due to the presence of 3-deoxyanthocyanin (carajurine) [2,3], have been widely exploited in the production of cosmetics [4]. In addition, the species *A. chica* has pharmacological properties, such as antimicrobial [5], anti-inflammatory, antiangiogenic and antiproliferative [6], wound healing [7], antiparasitic [8] and antioxidant properties [9,10,11]. However, although it is widely studied and its biological activities have already been described, there are still no studies on the biotechnological potential of metabolites produced by the endophytic microorganisms associated with *A. chica*.

Endophytic microorganisms are those that live inside plant tissues without causing harm to the host species. They have the ability to interact with the plant at complex levels and, due to this interaction, plants can modulate the metabolic process of these endophytes to produce molecules that have protective functions in relation to the microbe and the host [12].

In recent decades, endophytic fungi have gained prominence as a rich source of natural compounds with interesting pharmacological activities [13,14,15]. Therefore, exploring endophytic fungi that inhabit medicinal plants provides ample opportunities to discover new metabolites with potential bioactivity [16,17,18,19,20,21,22]. In addition, investigations regarding endophytes from tropical plants are still limited, especially when considering the pharmacological potential of these isolates [23].

Endophytic fungi are an unlimited source of new metabolites, and the use of crude extracts from these microorganisms may be a promising alternative since its bioactive compounds can be produced on an industrial scale, thus contributing to both a reduction in cost of the final product and the preservation of plant species [11]. In this sense, endophytes from plants that grow in special ecological niches, such as in the Amazon biome, may have the ability to produce a myriad of secondary metabolites. The bioactive substances resulting from the secondary metabolism of these microorganisms directly contribute to the adaptation of species and their survival, and they are often produced in stress situations [13,24,25]. Flavonoids, alkaloids, steroids, terpenoids, isocoumarins and phenols are among the classes of substances produced by endophytic fungi that present numerous biological activities such as hormonal, antitumor, cytotoxic, antiviral, immunosuppressive, antiparasitic, antimicrobial and antioxidant activities, among others [15,21].

Thus, considering the environmental conditions under which *A. chica* lives in the Amazon rainforest, such as high humidity, constant rainfall and high temperatures, as well as the presence of parasites and natural competitors, it is assumed that the endophytic fungi resident in this plant have the ability to produce bioactive substances to protect its host. Therefore, in this study, we evaluated the production of antimicrobial and antioxidant secondary metabolites produced by the endophytic fungi isolated from leaves and branches of the Amazonian species *A. chica* and identified the most promising fungal species as new sources of bioactive metabolites. This is the first report on the bioactivity of the metabolites of endophytic fungi that inhabit the aerial parts of *A. chica*. Furthermore, this study contributes to the increase in knowledge regarding the biodiversity of the Amazon.

## 2. Materials and Methods

### 2.1. Reagents

The materials consisted of culture media, bacterial and fungal strains, commercial antibiotics as well as analytical grade reagents. Potato dextrose agar (PDA) and Mueller–Hinton broth were purchased from Kasvi (Kasvi, São José dos Pinhais, Brazil). Sabouraud broth and yeast extract were supplied by Himedia (Himedia, Thane, India). Microorganisms’ strains were acquired from Cefar (Cefar Diagnóstica, Jardim Taquaral, Brazil). Levofloxacin, terbinafine and chloramphenicol were obtained from EMS (EMS pharma, Hortolândia, Brazil). Dextrose, methanol, ethyl acetate, FeCl_3_, dimethyl sulfoxide (DMSO), 2,3,5-triphenyltetrazoic chloride (TTC) and Folin–Ciocalteu solution were purchased from Dinâmica (Dinâmica, Indaiatuba, Brazil). Resazurin, 2,2-diphenyl-1-picrylhydrazyl (DPPH), quercetin, ascorbic acid, Trolox and gallic acid were obtained from Sigma-Aldrich (Sigma-Aldrich, Saint Louis, MO, USA). 2,4,6-Tripyridyl-s-triazine (TPTZ) was supplied by Merck (Merck, Darmstadt, Germany).

### 2.2. Endophytic Fungi

The endophytic fungi used in this study were isolated from the aerial parts of three shrubs of *A. chica* (variety II), which were obtained in February 2019 from Embrapa Western Amazon, located on highway AM-010, KM 29 (Manaus–Itacoatiara highway). The plant exsiccate was deposited in the herbarium of the Amazonas Federal Institute (IFAM), under code EAFM2901.

Samples of branches and leaves from the three specimens were washed with detergent under tap water, fragmented into 10 × 12 cm pieces and subjected to a sequence of submersions in different solutions in the following order and times: (i) for the leaves, 70% alcohol for 1 min; sodium hypochlorite 3% for 2.5 min, 70% alcohol for 30 s and sterile distilled water for 2 min; (ii) for the branches, 70% alcohol for 1 min; sodium hypochlorite 4% for 3 min, 70% alcohol for 30 s and sterile distilled water for 2 min [26].

For the isolation of endophytic fungi, the plant material was cut into pieces of approximately 6 mm^2^ and inoculated in Petri dishes (6 fragments in each plate) containing PDA with 50 mg/mL of chloramphenicol added. The plates were incubated at 26 °C for 15 days. According to the cultivable endophytes that had been grown, these were transferred to inclined test tubes containing PDA medium [26]. The isolated fungi were deposited in the Central Microbiological Collection (CCM) of the Amazonas State University (UEA). Access to genetic heritage was registered in the National System for the Management of Genetic Heritage and Associated Traditional Knowledge under code A0B4857.

One hundred and seven fungi, stored using the Castellani [27] method, were reactivated by inoculating a fragment of the stock culture in Petri dishes containing PDA, with subsequent incubation in a microbiological chamber (BOD) at 28 °C for 5–7 days. Table 1 presents information on the 107 endophytic fungi used in the present study for assessing their antioxidant and antimicrobial activity.

### 2.3. Production of Fungal Metabolites

Three fungal mycelium fragments (5 × 5 mm in diameter) that were removed from the PDA plates were inoculated into 250 mL Erlenmeyer flasks with 150 mL of liquid medium of the following composition: white potato broth (200 g/L); dextrose (10 g/L); yeast extract (2.0 g/L); NaCl (5.0 g/L); pH 5.0. The cultures were carried out under static conditions at 30 °C for 14 days, according to the methodology of Bose et al. [28], with modifications.

After cultivation, the metabolites were extracted with ethyl acetate in a 1:1 ratio for 4 h at room temperature and shaking at 120 rpm. After this period, the mycelium was removed via filtration in a Büchner funnel, and the fractions were separated in a separation funnel. The solvent was evaporated via the fume hood and the extracts were resuspended at a concentration of 10 mg/mL with a 10% DMSO solution and frozen (−18 °C) for later use in the biological tests [29].

### 2.4. Antimicrobial Testing

The microdilution technique was used according to the Clinical and Laboratory Standards Institute (CLSI) [30], which involved reducing resazurin for the antibacterial tests and reducing TTC for the antifungal tests. For the preliminary screening, fungal metabolites were tested against commercially acquired strains: *Staphylococcus aureus* CCCD-S009, *Escherichia coli* CCCD-E005 and *Candida albicans* CCCD-CC001. The extracts that showed activity against at least one of the two bacteria tested were evaluated against other strains: *Pseudomonas aeruginosa* CCCD-P004, *Proteus mirabilis* CCCD-P001, *Bacillus subtilis* CCCD-B005, *Staphylococcus epidermidis* CCCD-S010, *Enterococcus faecalis* CCCD-E002, *Serratia marcescens* CCCD-S005, *Klebsiella pneumoniae* CCCD-K003 and *Salmonella enterica* CCCD-S003. For the extracts that showed activity against *C. albicans*, these were also tested against *C. tropicalis* CCCD-CC002, *C. parapsilosis* CCCD-CC004 and *Aspergillus brasiliensis* CCCD-AA001.

For the test, 96-well microplates were used, which contained 100 µL of the extract at different concentrations (10, 5.0, 2.5, 1.25, 0.625 and 0.312 mg/mL) and 100 µL of the microbial inoculum. The microbial inoculum was prepared from colonies grown for 24 h. The microbial suspension was standardized at 0.5 on the McFarland Scale (10^8^ CFU/mL) and diluted in the culture medium (Mueller–Hinton broth for the bacteria and Sabouraud broth for the fungi) until reaching 5 × 10^5^ CFU/mL.

The positive control used for the bacteria was levofloxacin at 0.25 mg/mL and, for the fungi, terbinafine was used at 0.40 mg/mL. As a negative control, only the microbial inoculum was inserted and, for sterility control, 100 µL of the sterile culture medium that was used for the preparation of the inoculum was placed in the wells. A blank test was also performed, containing DMSO at different concentrations (1–100%), to ensure that the solvent used to dilute the extracts (DMSO 10%) did not present antimicrobial activity.

Subsequently, the plates were incubated at 37 °C for 24 h (bacteria) and 48 h (fungi) in a BOD chamber. After adding 30 μL resazurin at 0.01% or TTC at 1%, the plates were incubated again at 37 °C for 1–2 h to verify the change in color as a result of the reduction of the dyeing reagents.

For the extracts that showed activity, the minimum inhibitory concentration (MIC) was determined by successive dilutions of the samples. The lowest concentration of the extracts that inhibited microbial growth was considered the MIC.

### 2.5. Antioxidant Assays

Antioxidant activity was determined using the 2,2-diphenyl-1-picrylhydrazyl radical sequestration (DPPH•) method. The DPPH• solution was prepared at a concentration of 0.06 mmol/L, with methanol P.A., protected from exposure to direct light [31]. The assay was performed in 96-well microplates, with 40 µL of extract and the addition of 250 µL of the DPPH• solution. For the negative control, 40 µL of 10% DMSO and 250 µL of DPPH• solution were added [32]. The microplate was protected from exposure to direct light and, after 10 min, the absorbance readings were performed in a microplate reader (Molecular Devices, Spectramax Plus) at 517 nm. Experiments were performed in triplicate.

Fungal extracts were first tested at a single concentration of 10 mg/mL. Quercetin was used as the standard, at a concentration of 40 µg/mL. The percentage of sequestration of DPPH• radicals was calculated using Equation (1), using the values of the absorbance decay of the sample (Abs_sample_) and the control (Abs_control_):

(1) $$\mathrm{AA}\;\left(\%\right)=\frac{{\mathrm{Abs}}_{\mathrm{control}}-{\mathrm{Abs}}_{\mathrm{sample}}}{{\mathrm{Abs}}_{\mathrm{control}}}\times 100$$

For the samples that showed activity (AA > 70%), the efficient concentration for the sequestration of 50% of the DPPH• radicals (EC_50_) was determined, which was calculated from the successive dilutions of the samples and the generation of a linear regression graph. Extracts were evaluated in a concentration range from 10,000 to 4.88 μg/mL. Quercetin was tested from 100 to 3.125 μg/mL.

The antioxidant activity was also tested via the ferric reducing antioxidant power (FRAP) method, as described by Benzie and Strain [33] with modifications. The FRAP reagent consists of 100 mL of acetate buffer (0.3 mM), 10 mL of TPTZ (10 mM) and 10 mL of aqueous solution of ferric chloride (20 mM). To test the antioxidant activity, 2.45 mL of the FRAP reagent was incubated with 0.35 mL of the sample for 30 min, at 37 °C, under protection from light. The samples were then analyzed in a spectrophotometer UV–Vis (UV 1800, Shimadzu, Kyoto, Japan) at 595 nm. All experiments were performed in triplicate.

Samples of the extracts were tested at a single concentration of 10 mg/mL. A standard curve was constructed with Trolox in 10% DMSO. The results were expressed in μmol of Trolox equivalent per g of extract (μmol TE/g). Ascorbic acid was used as the standard, at a concentration of 40 µg/mL. DMSO 10% was used as a blank.

### 2.6. Chemical Profile of Fungal Extracts

The fungal extracts considered most promising in relation to biological activity tests were subjected to analysis via thin layer chromatography (TLC) in order to identify the main chemical classes present in the active metabolites. Then, 20 mg of the extract was solubilized in 2 mL of methanol. With the aid of capillaries, 2 μL of the samples were placed on a silica gel chromatographic plate (TLC aluminum sheets, Macherey-Nagel, 20 × 20 cm, silica gel 60 matrix, fluorescent indicator). Dichloromethane:acetone (9:1) was used as the mobile phase (10 mL volume).

To detect the chemical classes, ultraviolet light at 254 nm and 365 nm and the following chemical developers were used: *p*-anisaldehyde, ferric chloride, aluminum chloride and Dragendorff’s reagent. To prepare the *p*-anisaldehyde developer, 0.5 mL of *p*-anisaldehyde was mixed into 10 mL of acetic acid, 85 mL of methanol and 5 mL of concentrated H_2_SO_4_. The ferric chloride was obtained by diluting 3 g of FeCl_3_ in 100 mL of ethyl alcohol. Aluminum chloride was prepared with 1 g of AlCl_3_ in 100 mL of ethyl alcohol. Dragendorff’s reagent was prepared with 5 mL of Solution I (0.85 g of basic bismuth nitrate in 10 mL of glacial acetic acid added to 40 mL of distilled water) and 5 mL of Solution II (8 g of potassium iodide in 20 mL of distilled water), with the addition of 20 mL of acetic acid and distilled water to complete the volume to 100 mL [34].

### 2.7. Dosage of Total Phenolic Content

The samples that were considered most promising had their total phenolics measured using the Folin–Ciocalteu methodology, as described by Singleton and Rossi [35], with modifications. An aliquot of 0.25 mL of the fungal extract (10 mg/mL) and 2.75 mL of the 3% Folin–Ciocalteu solution were used. The samples were vortexed for 10 s, followed by 5 min of rest. After this period, 0.25 mL of the 10% Na_2_CO_3_ solution was added and the mixtures were incubated at room temperature, while protected from exposure to light for one hour. Subsequently, the absorbance was determined at 765 nm in a UV–Vis spectrometer (UV-1800, Shimadzu, Kyoto, Japan). A gallic acid solution at 200 mg/mL in ethanol was used to prepare the standard curve (0, 25, 50, 75, 100, 150 and 200 mg/mL) and ethanol was used as the blank. The gallic acid calibration curve was used to quantify the total phenolics. The results were expressed in equivalents of gallic acid per 100 mg of the extract (mg GAE/100 mg). All experiments were performed in triplicate.

### 2.8. Identification of the Most Promising Fungi

The fungi selected as being the most promising were identified by classical taxonomy, as well as using molecular tools. The macromorphological features were analyzed after the fungi were cultured during 7 days at 28 °C in Petri dishes (10 mm × 90 mm) containing PDA. The macroscopic vegetative characteristics, which were color, texture, topography, diffuse pigmentation, color, border and topography of the back of the colony were analyzed [36]. The micromorphology (hyphae and reproductive structures) was assessed using the microculture technique in PDA for 5–7 days. The macroscopic glass slides were stained with lactophenol blue and analyzed in an optical microscope (40×) [37]. The obtained results were compared with taxonomic keys [38,39]. 

The most promising fungi were also identified by sequencing the three DNA loci-internal transcribed spacer (ITS), β-tubulin (βtub) and calmodulin (CaM). The genomic DNA was extracted using the CTAB method [40], with modifications, following the protocol described by Oetari et al. [41], with modifications: DNA amplification via PCR (polymerase chain reaction) had a final reaction volume of 15 µL: 3 mM MgCl_2_, 0.2 mM dNTPs, 1× buffer 10×, 0.2 mM forward primer, 0.2 mM reverse primer, 1 U Taq polymerase and 50 ng fungal genomic DNA. The same protocol was used for each of the primers used in this study: ITS1 and ITS4 [42], βtub3 and βtub4r [43], CaM-228F and CaM-737R [44]. The amplification conditions consisted of the following steps: denaturation for 5 min at 95 °C; annealing with 35 cycles at 95 °C, for 30 s, 35 cycles at 54 °C (CaM) or 62 °C (ITS and βtub), depending on the specific hybridization temperature for each primer, for 30 s, followed by 35 cycles at 72 °C, for 1 min and a final extension at 72 °C, for 5 min. The same cycling conditions were used for all primers. The amplified product was subjected to an electrophoretic run on 1.5% agarose gel to verify its efficiency.

The amplified PCR product was purified with PEG 8000 20% and the sequences were read in an automatic sequencer (ABI 3130xl Genetic Analyzer, Applied Biosystems, Thermo Fisher, Waltham, MA, USA). The sequences were manually checked, aligned, edited and analyzed with the help of Bioedit v.7.2.6 [45]. As the reference standards, we used the sequences deposited in GenBank (to obtain the preliminary identification) and the sequences with high similarity with the type specimens for the phylogenetic analysis. The sequences obtained were deposited in GenBank. For the construction of the genetic tree, the sequences of the combined loci (ITS + βtub + CaM) were aligned using the MAFFT program version 7 “https://mafft.cbrc.jp/alignment/software/ (accessed on 10 April 2023)” and phylogenetic analyses were conducted via the MEGA program version X [46], using the maximum-likelihood method, with 1000 bootstrap replicates.

### 2.9. Statistical Analysis

The experimental data obtained from antioxidant activity and total phenolic content were submitted to analysis of variance (ANOVA) for homogeneous samples; and to the Bonferroni test with a 95% confidence interval to distinguish the differences. The software used was BioEstat v. 5.0.

## 3. Results

### 3.1. Antimicrobial Activity of Fungal Extracts

The metabolic extracts of the 107 endophytic fungi isolated from *A. chica* were evaluated for antimicrobial activity. Of the total extracts evaluated, 18 showed activity against at least one of the microbial strains tested (Table 2). The fungus CF2-13, isolated from the *A. chica* leaves, was the most promising regarding the production of metabolites with antimicrobial activity. This fungus was able to inhibit the growth of Gram-negative (*E. coli*; *S. enterica*, *P. mirabilis*, *K. pneumoniae* and *S. marcescens*) and Gram-positive (*S. aureus*, *S. epidermidis* and *B. subtilis*) bacteria; as well as fungi (*C. albicans*, *C. tropicalis*, *C. parapsilosis* and *A. brasiliensis*).

### 3.2. Antioxidant Activity of Fungal Extracts

Of the fungal extracts evaluated at 10 mg/mL, 70 showed antioxidant activity (AA) over 70% and were considered active (Table 3). The ANOVA results indicated that there are significant differences (*p* < 0.05) between the mean values of AA. Of the 70 active extracts, 11 showed the highest AA values, between 97.75 and 100%, without statistical difference between them and when compared to the quercetin reference standard at 40 μg/mL (*p* < 0.05).

Seven extracts showed an EC_50_ of <1000 μg/mL, which can be considered as promising in the case of crude extracts. The metabolites produced by the fungus CG1-7, isolated from the branches of *A. chica*, presented the lowest EC_50_ value, and were able to sequester 50% of the DPPH• free radicals at a concentration of 11 μg/mL. This result is comparable to the EC_50_ value found for the quercetin reference standard (8 μg/mL). The isolate CF2-13, selected as the best producer of antimicrobial substances, presented an EC_50_ of 360 μg/mL.

The fungal extracts that presented AA of >70% were also evaluated via the FRAP method, which was used as a second indicator of antioxidant activity. The FRAP results were obtained from a calibration curve (y = 0.0034x − 0.0448, R^2^ = 0.9927) of Trolox (0–140 μM) and expressed in Trolox equivalents (TEs) per g of extract. The results of the ANOVA indicated that there is significant difference between the means of FRAP results (*p* < 0.05).

The results of the FRAP assay corroborate the antioxidant potential of the metabolites produced by the endophytic fungi isolated from *A. chica*. Of the 70 extracts evaluated, 20 presented higher antioxidant power (166.0–218.6 μmol TE/g), without statistic difference between them and when compared to the ascorbic acid reference standard (*p* < 0.05). The isolates CF1-13 and CF2-16, both from *A. chica* leaves, presented the highest FRAP values (>200 μmol TE/g). However, these two endophytic fungi had an EC_50_ > 1000 μg/mL, i.e., low DPPH free-radical scavenging potential. The endophytes CF1-37, CG2-10, CF3-5 and CG3-7 presented promising results for both antioxidant methods (DPPH and FRAP) and should be further investigated in order to explore its production of metabolites since they may be new sources of antioxidant molecules. Thus, since the EC_50_ was considered the main parameter in our screening for antioxidant activity, the fungus CG1-7 was selected.

### 3.3. Chemical Profile of Promising Fungal Extracts

**Figure 1:**
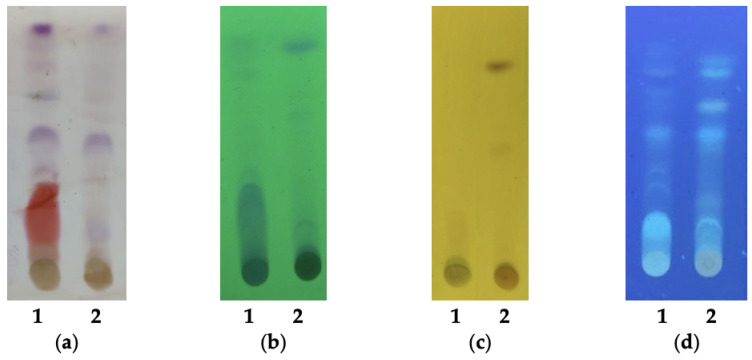
Thin layer chromatography of the metabolic extracts of endophytic fungi from *Arrabidaea chica* CG1-7 (1) and CF2-13 (2). (**a**) Stained with *p*-anisaldehyde, indicating the presence of terpenes (purple spots) and flavonoids (red spot); (**b**) UV light at 254 nm, indicating the presence of conjugated double bonds; (**c**) stained with ferric chloride, indicating the presence of phenolic compounds; (**d**) stained with aluminum chloride and exposure under UV light at 365 nm, indicating the presence of flavonoids.

The extracts of the fungi CF2-13 and CG1-7 were analyzed using TLC in order to identify the chemical classes present in the active extracts. After staining the chromatographic plates with *p*-anisaldehyde, purple spots were observed in both extracts, which indicate the presence of terpenes; and a red intense spot in the CG1-7 extract, which indicates the presence of flavonoids (Figure 1a). The visualization of the chromatographic plates under UV light at 254 nm revealed spots that indicate the presence of conjugated double bonds (Figure 1b). The brown spot on the CF2-13 sample, when developed with ferric chloride, indicates the presence of phenolic compounds (Figure 1c) and the fluorescence, when the samples were treated with aluminum chloride and exposed to UV light at 365 nm, indicating the presence of flavonoids in both extracts (Figure 1d). The presence of alkaloids in the bioactive extracts was not detected in the TLC.

### 3.4. Total Phenolic Content of the Most-Active Fungal Extracts

Phenolic compounds are important secondary metabolites with redox properties that are responsible for antioxidant activity. The total phenolic content was measured in the metabolic extracts produced by the endophytic fungi CF2-13 and CG1-7. The total phenolic results were obtained from a calibration curve (y = 0.0035x − 0.0403, R^2^ = 0.9901) of gallic acid (0–100 µg/mL) and expressed in gallic acid equivalents (GAEs) per 100 mg of extract.

The content of phenolic compounds in bioactive extracts of CF2-13 and GC1-7 was found to be 3.70 and 5.28 mg GAE/100 mg of extract, respectively. The extract produced by CG1-7 presented a higher concentration of phenolic compounds than the extract produced by CF2-13 (*p* < 0.05), which is in accordance with the antioxidant activity results.

### 3.5. Identification of Endophytic Fungi That Produce Bioactive Substances

**Figure 2:**
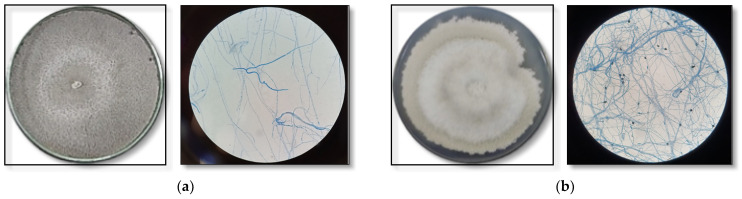
Macro- and micromorphological characteristics of endophytic fungi from *Arrabidaea chica* whose metabolic extracts showed the most-promising biological activities: antimicrobial-*Botryosphaeria mamane* CF2-13 (**a**); and antioxidant-*Colletotrichum* sp. CG1-7 (**b**).

The fungi that produced the most-promising bioactive extracts were identified using classical taxonomy. The macro and micromorphological characteristics of the most-promising isolates can be seen in Figure 2.

The endophytic fungus CF2-13 isolated from the leaves of *A. chica* showed a colony of rapid vegetative growth that was cottony, irregular, flat, initially creamy-white in color, darkening with the aging of the inoculum to lead-gray, with a dark reverse side, moderately dense mycelia culture and slow sporulation. Micromorphological characteristics: septate, hyaline hyphae; ellipsoid, fusiform conidia with both ends straight, smooth-walled, hyaline, aseptate and scarce (Figure 2a).

The endophytic fungus CG1-7 isolated from the branches of *A. chica* presented a colony with fast vegetative growth that was cottony, irregular, flat, whitish to gray towards the center, with a dark reverse side, and dense mycelia culture, but slow sporulation. The micromorphological characteristics include septate, hyaline, thin and dense hyphae; cylindrical, fusiform conidia with both obtuse ends, smooth-walled, hyaline, aseptate and scarce; presence of appressoria (abundant) globose, some clavate, complex with irregular lobes, aseptate and brown in color (Figure 2b).

These fungal isolates were subjected to molecular identification using combined loci (ITS + βtub + CaM) to determine their identity. The isolate CF2-13, obtained from the leaves of *A. chica*, was identified with 99% of maximum likelihood as *Botryosphaeria mamane* (= *Cophinforma mamane*) from the combined analysis (ITS + βtub). However, for this isolate, there was no resolution for the calmodulin locus.

The fungus CG1-7, obtained from *A. chica* branches, was identified with 82% of maximum likelihood as *Colletotrichum siamense* from the combined analysis (ITS + βtub + CaM). Considering that the maximum likelihood was 82%, the fungal species was not confirmed and, therefore, other locus should be used for proper molecular identification.

**Figure 3:**
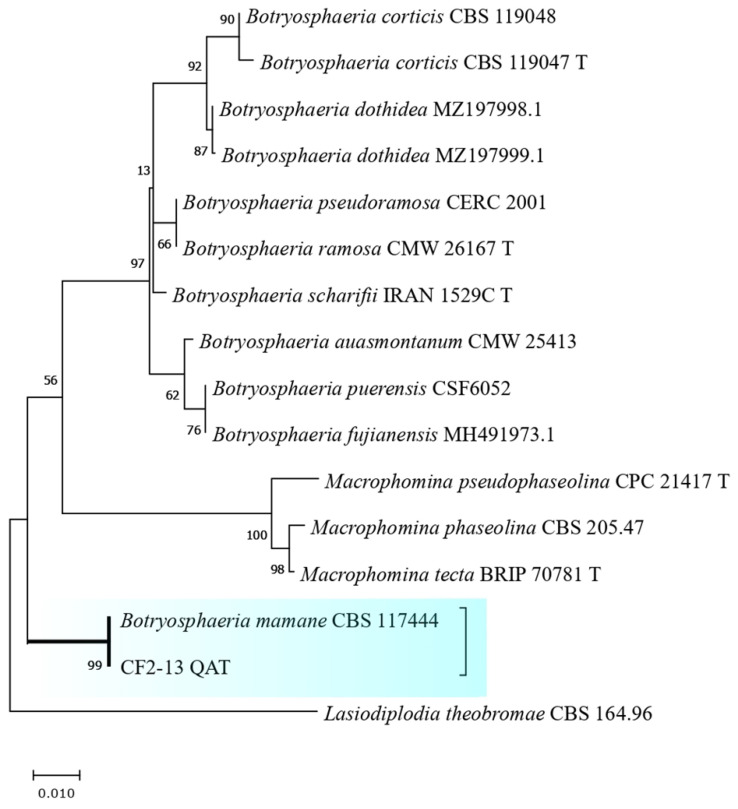
Combined phylogeny of the endophytic fungus CF2-13 isolated from *Arrabidaea chica* leaves using ITS and β-tubulin. The branch with the thicker line indicates the isolate that was sequenced in this study. The scale bar indicates nucleotide substitutions per site, using the neighbor-joining method via maximum-likelihood analysis. The numbers of the nodes indicate the bootstrap values of 1000 replicates. The tree was rooted in *Lasiodiplodia theobromae* CBS 164.96.

**Figure 4:**
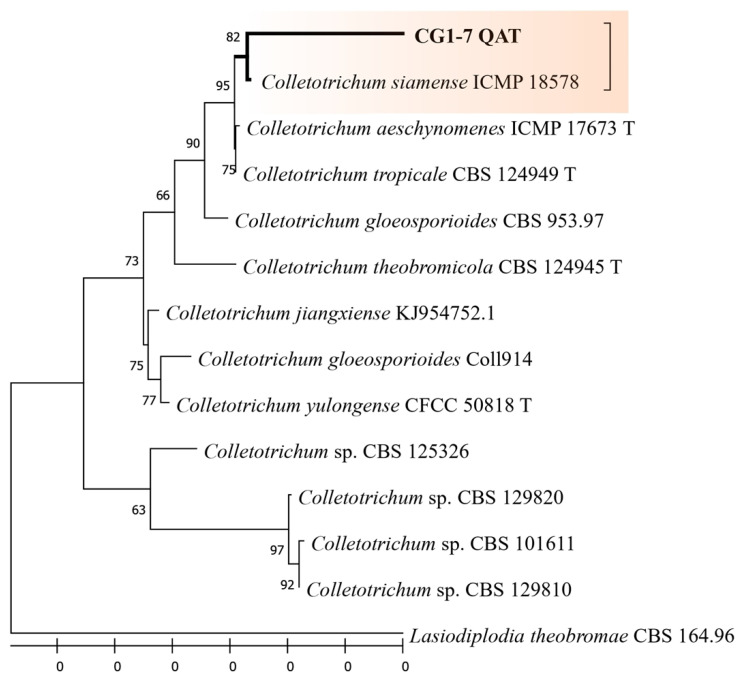
Combined phylogeny of the endophytic fungus CG1-7 isolated from *Arrabidaea chica* branches using ITS, β-tubulin and CaM. The branch with the thicker line indicates the isolate that was sequenced in this study. The scale bar indicates nucleotide substitutions per site, using the neighbor-joining method via maximum-likelihood analysis. The numbers of nodes indicate the bootstrap values of 1000 replicates. The tree was rooted in *Lasiodiplodia theobromae* CBS 164.96.

Figure 3 and Figure 4 show the result of the phylogenetic trees obtained for the isolates *B. mamane* CF2-13 and *Colletotrichum* sp. CG1-7, respectively, revealing the evolutionary history of the sequences analyzed using the maximum-likelihood method and the Tamur–Nei model. The sequences obtained in this study were deposited in GenBank (Table 4).

## 4. Discussion

The biological/biochemical role of endophytic fungi in a plant and how they interact with the host and with other endophytes and organisms associated with the plant species is still unclear [13,47]. However, the microbial diversity that different plant species harbor, together with the chemodiversity of the metabolites that endophytic fungi produce, offer the opportunity for the discovery of new bioactive molecules with different biotechnological applications [21]. In addition, several studies have demonstrated the usefulness of endophytic microorganisms in host survival, since endophytes directly influence plant metabolism in order to, for example, resist extreme temperatures and periods of drought, as well as the presence of phytopathogens [48]. Therefore, the traditional use of the plant and the region in which it inhabits are important criteria to be considered for the isolation of endophytes [49,50].

The species *A. chica* is widely cultivated in the Amazon and its leaves are traditionally used as astringents, genital disinfectants, in the treatment of inflammations, skin diseases and wound healing, intestinal colic, dysentery, leukorrhea and anemia [51], and it has proven antimicrobial and antioxidant activity [5,9,11]. Thus, taking into account that the need for new compounds with antimicrobial potential and antioxidant activity is inevitable, we studied the population of endophytic fungi of *A. chica* for its antimicrobial and antioxidant potential.

The antimicrobial activity of extracts of endophytic fungi isolated from *A. chica* was investigated against strains of bacteria and fungi known to be pathogenic to humans. Of the total number of extracts evaluated, 19% inhibited the growth of at least two pathogens. We noticed that the fungi isolated from the leaves of *A. chica* proved to be more promising regarding antimicrobial activity, when compared to endophytes isolated from the branches. Eleven isolates from the leaves produced antimicrobial metabolites, while only seven fungi from the branches showed activity. These results are in accordance with those observed by Santos et al. [50]. The authors found that endophytic fungi from the leaves of *I. suffruticosa* produced bioactive metabolites with antimicrobial activity.

It was observed that the extract of the fungus identified as *Botryosphaeria mamane* CF2-13 exhibited the best antimicrobial activity against the bacteria *S. aureus* and the yeast *C. parapsilosis* (MIC = 0.325 mg/mL). The species *B. mamane* was first described by Gardner [52] as a phytopathogen, associated with witches’ brooms on *Sophora chrysophylla*, in Hawai. However, this species has also been isolated as an endophyte and some studies demonstrate the potential of its bioactive metabolites. *B. mamane* was isolated as an endophyte of *Garcinia mangostana* and showed promise as a producer of metabolites with antimicrobial activity against *S. aureus* and methicillin-resistant *S. aureus* [53], thus corroborating the results of the present study. In another study, Oliveira et al. [54] identified volatile organic compounds produced by the endophyte *B. mamane* isolated from plants collected in the Caatinga biome of northeastern Brazil. More recently, Triastuti et al. [55] used *B. mamane* isolated from the medicinal plant *Bixa orellana* L. to observe how histone diacetylase inhibitors alter the production of its secondary metabolites. This fungal species has been renamed *Cophinforma mamane* [56] (*Botryosphaeria mamane* D.E. Gardner = *Cophinforma mamane* (D.E. Gardner) A. J. L. Phillips and A. Alves) and is considered a rich source of new bioactive substances, though it is still poorly studied for biotechnological applications.

Other species of *Botryosphaeria* isolated as endophytes also have antimicrobial potential. Silva et al. [22] evaluated the antimicrobial activity of metabolites from *B. fabicerciana* that were isolated from *Morus nigra* L. and observed an MIC of 64 µg/mL for *S. aureus*, and an MIC of 1000 µg/mL for *E. coli*. Xiao et al. [57] identified 17 of the metabolites produced by *B. dothidea*. The substance pycnophorin, which is produced by the endophyte, inhibited the growth of *B. subtilis* and *S. aureus*, with an MIC of 25 µM; while stemphyperylenol demonstrated high antifungal activity against the phytopathogen *Alternaria solani* (MIC = 1.57 µM). It has been observed that fungi belonging to this genus produce exometabolites such as jasmonic acid and its derivatives, polyketides such as lasiodiplodin and isocofumarin, chaetoglobosins and alternariol analogues, among others, with potential bioactivity [57,58,59,60]. These findings justify the investigation of species of this genus as possible sources of antimicrobial-producing fungi and also corroborate the results presented in this work.

In the present study, *B. mamane* CF2-13 produced metabolites with promising antifungal activity, especially for yeasts of the genus *Candida*. *C. albicans* is the most common fungal pathogen in humans; it causes invasive infections and is a serious problem, especially in immunosuppressed patients. However, the epidemiology of fungal infections is evolving rapidly. Other *Candida* species have emerged as the main opportunistic pathogens, are associated with oral mucosa and have been identified as being commensal for a minority of healthy individuals [61]. The rise of the multidrug-resistant fungal pathogen *C. auris*, for instance, poses a global public health menace, and has gained significant attention for its swift and extensive proliferation in the last decade [62]. The anti-*Candida* activity of metabolites of the endophyte *B. mamane* CF2-13, therefore, is worthy of further investigation as a new source of antifungal substances. Additionally, these findings agree with what has been reported for the active metabolites of the host plant [5,63], and for its popular use [50]. According to Matos [64], indigenous tribes in the Amazon use a decoction of *A. chica* leaves for treating fungal infections and for cleaning chronic wounds.

Free radicals are known to induce oxidative damage to the body and, consequently, cause various disorders such as cancer, heart disease, Parkinson’s, Alzheimer’s, cataracts, diabetes mellitus, arthritis and premature aging [65,66]. Several studies have shown that both medicinal plants and their endophytes can be a potential source of molecules with antioxidant activity [2,14,20]. Some authors even suggest that the medicinal properties of the host plant may be a consequence of the capacity of its endophytic microorganisms to produce biologically active secondary metabolites [50]. The plant *A. chica* is known as a producer of antioxidant substances [9,10,11]. Our study demonstrates that *A. chica* endophytic fungi are also a promising source of antioxidant compounds.

The screening of extracts produced by *A. chica* endophytic fungi for free-radical scavenging activity via the DPPH• assay showed that, of the extracts evaluated, 65% have potential antioxidant activity, with AA > 70%. In terms of antimicrobial activity, the most promising metabolites for antioxidant activity were produced by isolates from *A. chica* leaves, when compared to the isolates obtained from the branches. These results indicate that there is a more pronounced production of bioactive secondary metabolites in fungi associated with the leaves of *A. chica*, and this could be explained by the metabolites that are produced in *A. chica* leaves. Siraichi et al. [9] identified the phenolic compounds isoscutellarein, 6-hydroxyluteolin, hispidulin, scutellarein, luteolin and apigenin in *A. chica* leaves, and attributed the significant antioxidant activity found in the extract obtained from its leaves to the presence of the mixture of flavonoids, with the main contribution being from scutellarein and apigenin.

The extract of the fungus identified as *Colletotrichum* sp. CG1-7 exhibited the strongest antioxidant activity, with the same potency obtained for the antioxidant reference standard quercetin. The extract of the fungus *B. mamane* GF2-13, selected as the most promising producer of antimicrobial substances, also presented radical scavenging action and showed itself to be a potential new source of bioactive compounds. The genus *Colletotrichum* is one of the most commonly isolated as an endophyte [13]. Despite being known to cause anthracnose in cereals, vegetables and fruit trees, fungi of this genus produce a variety of bioactive secondary metabolites. Molecules containing nitrogen, sterols, terpenes, pyrones, phenolics and fatty acids have been identified among the metabolites of these fungi [67]. Chithra et al. [68] isolated piperine, a substance considered a potent antioxidant, from the metabolites of the endophytic fungus *C. gloeosporioides* isolated from *Piper nigrum*. Tianpanich et al. [69] found that two isocoumarins produced by the endophytic fungus *Colletotrichum* sp. eliminated DPPH• free radicals (EC_50_ of 23.4 and 16.4 µM). On the other hand, contrary to what was observed in the present study with the isolate of *A. chica*, Mahmud et al. [70] observed the low antioxidant activity of the extract obtained from *C. siamense* fungus, which was isolated from *Justicia gendarussa*. The different methods of obtaining the fungal extract, as well as the different host, its location, climatic conditions and the isolation site, may explain the difference in the bioactivity of the metabolites of endophytic fungi.

Several studies have demonstrated the antioxidant potential of endophytic fungi. Budiono et al. [71] observed the antioxidant activity of metabolites from the endophytic fungi of *Syzygium samarangense* L., with the fungus *Lasiodiplodia venezuelensis*, which was isolated from leaves and was shown to be the most promising (EC_50_ = 49.96 µg/mL for the fungal extract). Alves et al. [72] evaluated the activity of endophytic fungi isolated from *Jatropha curcas* L. and the species *Aspergillus nidulans* presented highly promising metabolites in DPPH• sequestration, with an EC_50_ of 5.40 µg/mL. Druzian et al. [73] evaluated the extracts of metabolites produced by the species *Botryosphaeria dothidea* and obtained an EC_50_ of 206 μg/mL. Thus, it can be claimed that the endophytes of *A. chica* are promising sources of antioxidant substances, since seven isolates presented an EC_50_ of < 1000 μg/mL in their metabolic extracts.

The antioxidant potential of extracts from *A. chica* endophytic fungi was also evaluated using the FRAP method. The fungal extracts showed themselves to be able to convert Fe^3+^-tripyridyltriazine into Fe^2+^-tripyridyltriazine. The isolates CG2-13 and CF2-16 showed promising FRAP results; however, their metabolites were not effective in the DPPH scavenging activity assay. Pulido et al. [74] suggest that the FRAP test is a useful tool for studying the antioxidant effectiveness of various extracts and pure substances. However, this test may not accurately reflect the process of radical elimination in lipid systems and may not correlate well with other measurements of antioxidant activity. Therefore, it is recommended to combine the FRAP test with other methods in order to better understand the dominant mechanisms of different antioxidants [75].

The DPPH test and FRAP test are often applied in antioxidant investigation, with sets of experiments linked to electrons or radical scavenging. They work based on the reduction process. The DPPH test is used to estimate the antioxidant activity based on the process through which antioxidants limit lipid oxidation, resulting in DPPH free-radical scavenging and therefore determining free-radical scavenging potential. The ferric reducing antioxidant method is performed on electron-transfer processes wherein a ferric salt is applied as an oxidant. The oxidation of ferric 2,4,6-tripyridyl-s-triazine to the colorful ferrous state is the reaction mechanism [76].

According to Aguirre et al. [77], some of the natural antioxidant compounds abundantly produced by endophytic fungi are from the class of phenolic compounds, such as flavonoids and phenolic acids, which corroborates the results found in this study. The TLC results indicate the presence of these classes of molecules in the extracts produced by the two most promising isolates. The studies by Kaur et al. [78], for example, also observed the presence of phenolic compounds and flavonoids in the metabolic extracts of the endophytic fungus *Aspergillus fumigatus* isolated from *Moringa oleifera*, which showed pronounced antioxidant activity against DPPH• radicals (EC_50_ = 40.07 µg/mL).

The indications of the chemical classes observed in the extracts through the TLC analyses were dictated by the culture medium used in the growth of the fungi. The potato dextrose medium is a rich source of glucose, which in turn is a fundamental substrate in the biochemical pathways of shikimate and acetyl-CoA pathways that participate in the formation of terpenes and phenolic substances [79]. Another factor that enables the production of secondary metabolites was the addition of yeast extract to the medium, since this supplement is rich in vitamin B2, a component that participates in the synthesis of different secondary metabolites [80].

Several studies show that some species, including *B. mamane*, which belongs to the genus *Botryosphaeria*, when cultivated in a culture medium with potato, produce phenolic molecules with different structures [81,82]. In the study performed by Oliveira et al. [54], the fungi of the Botryosphaeriaceae family, including *B. mamane,* was evaluated regarding the production of volatile substances such as terpenes. The authors found that, when using potato medium as a substrate, most of the substances produced by the fungi were sesquiterpenes. Similarly, species of the genus *Colletotrichum*, when cultivated in potato as the carbon source, present a secondary metabolism that is mainly conditioned to the production of terpenes and phenolic molecules [67,83]. In our study, potato broth was used to produce the secondary metabolites of *A. chica* endophytic fungi, and therefore, we also found the production of phenolic compounds and terpenes in the TLC analysis.

Microbial sources are rich in phenolic compounds and these metabolites can be obtained via controlled conditions, and at faster speeds than when obtained from plants, which is an advantage in terms of production costs [84]. The optimization of *A. chica* endophytic fungi cultivation, in order to improve the production of bioactive phenolic compounds, could provide a higher concentration of bioactive molecules, and, therefore, should be pursued in future experiments.

## 5. Conclusions

This study provides information on the endophytic fungi isolated from the Amazonian species *A. chica*. This is the first report on the bioactivity of the metabolites of endophytic fungi that inhabit the aerial parts of this medicinal plant. The secondary metabolites produced by the *A. chica* endophytic fungi may contain novel and unexplored bioactive compounds. The data obtained show that, of the 107 extracts of endophytic fungi evaluated for antimicrobial and antioxidant potential, the extract of *B. mamane* CF2-13 exhibited significant antimicrobial potential against Gram-positive and Gram-negative bacteria, as well as against fungi. The extract of the isolate *Colletotrichum* sp. CG1-7 had pronounced antioxidant activity, which was equivalent to the reference standard. Both active extracts showed the presence of flavonoids. Further studies aiming at the structural elucidation of the molecules present in bioactive extracts should therefore be carried out.

## Figures and Tables

**Table 1:** Endophytic fungi isolated from the leaves and branches of the three specimens of *Arrabidaea chica* used in this study.

Endophytic Fungus Code				Specimen	Plant Part	Number of Isolates
CF1-1	CF1-12	CF1-22	CF1-31	1	Leaves	33
CF1-2	CF1-13	CF1-23	CF1-32			
CF1-3	CF1-15	CF1-24	CF1-34			
CF1-4	CF1-16	CF1-25	CF1-35			
CF1-5	CF1-17	CF1-26	CF1-36			
CF1-6	CF1-18	CF1-27	CF1-37			
CF1-7	CF1-19	CF1-28				
CF1-9	CF1-20	CF1-29				
CF1-11	CF1-21	CF1-30				
CG1-1	CG1-5	CG1-9	CG1-12	1	Branches	11
CG1-2	CG1-7	CG1-10	CG1-14			
CG1-4	CG1-8	CG1-11				
CF2-1	CF2-7	CF2-13	CF2-17	2	Leaves	16
CF2-2	CF2-9	CF2-14	CF2-18			
CF2-3	CF2-11	CF2-15	CF2-19			
CF2-6	CF2-12	CF2-16	CF2-20			
CG2-2	CG2-5	CG2-10	CG2-15	2	Branches	11
CG2-3	CG2-7	CG2-11	CG2-16			
CG2-4	CG2-8	CG2-12				
CF3-1	CF3-9	CF3-15	CF3-21	3	Leaves	22
CF3-2	CF3-10	CF3-16	CF3-23			
CF3-4	CF3-11	CF3-17	CF3-24			
CF3-5	CF3-13	CF3-18	CF3-26			
CF3-6	CF3-12	CF3-19				
CF3-7	CF3-14	CF3-20				
CG3-1	CG3-8	CG3-13	CG3-18	3	Branches	14
CG3-3	CG3-10	CG3-15	CG3-19			
CG3-4	CG3-11	CG3-16				
CG3-7	CG3-12	CG3-17				
					Total	107

**Table 2:** Minimum inhibitory concentrations (MICs, mg/mL) of the extracts of endophytic fungi from *Arrabidaea chica* that presented antimicrobial activity.

Endophytic Fungi Code	MIC (mg/mL)													
	EC	SA	CA	PA	PM	BS	SEp	EF	SM	KP	Sen	CT	CP	AB
CF1-2	5.00	-	-	-	-	5.00	-	-	-	-	NT	NT	NT	NT
CF1-26	5.00	-	-	-	-	-	-	-	-	-	5.00	NT	NT	NT
CF1-29	5.00	-	-	-	-	-	-	-	-	-	2.50	NT	NT	NT
CF1-37	-	5.00	1.25	-	-	-	-	-	-	-	-	1.25	2.50	-
CG1-10	-	5.00	-	-	-	5.00	-	5.00	-	-	2.50	NT	NT	NT
CG1-1	5.00	1.25	1.25	5.00	5.00	-	-	-	-	-	-	5.00	5.00	-
CG1-2	-	5.00	-	5.00	5.00	-	-	-	2.50	5.00	5.00	NT	NT	NT
CG1-5	5.00	5.00	-	-	-	-	-	5.00	-	-	5.00	NT	NT	NT
CG1-8	5.00	-	-	-	-	5.00	5.00	5.00	-	5.00	5.00	NT	NT	NT
CG1-9	5.00	-	-	-	-	-	5.00	5.00	-	-	-	NT	NT	NT
CF2-11	-	5.00	2.50	-	-	2.50	1.25	-	-	-	-	5.00	5.00	-
**CF2-13**	**2.50**	**0.312**	**1.25**	**-**	**5.00**	**2.50**	**1.25**	**-**	**5.00**	**5.00**	**2.50**	**1.25**	**0.312**	**2.50**
CF2-16	-	-	1.25	NT	NT	NT	NT	NT	NT	NT	NT	2.50	1.25	-
CG2-5	5.00	5.00	-	-	-	-	5.00	2.50	-	-	5.00	NT	NT	NT
CF3-5	-	-	1.25	NT	NT	NT	NT	NT	NT	NT	NT	2.50	1.25	-
CF3-9	-	5.00	-	NT	NT	NT	NT	NT	NT	NT	NT	NT	NT	NT
CF3-14	5.00	5.00	-	-	-	-	2.50	-	-	-	-	NT	NT	NT
CF3-26	-	-	5.00	NT	NT	NT	NT	NT	NT	NT	NT	5.00	5.00	-

EC = *Escherichia coli*; SA = *Staphylococcus aureus*; CA = *Candida albicans*; PA = *Pseudomonas aeruginosa*; PM = *Proteus mirabilis*; BS = *Bacillus subtilis*; SEp = *Staphylococcus epidermidis*; EF = *Enterococcus faecalis*; SM = *Serratia marcescens*; KP = *Klebsiella pneumoniae*; SEn = *Salmonella enterica*. CT = *Candida tropicalis*; CP = *Candida parapsilosis*; AB = *Aspergillus brasiliensis*; “-” = no antimicrobial activity; NT = Not tested. Levofloxacin was used as positive control for bacterial strains. MICs (μg/mL): EC = 0.05; SA = 0.50; PA = 0.50; PM = 0.25; BS = 0,50; Sep = 0.25; EF = 1.00; SM = 0.25; KP = 4.00; Sen = 0.25. Terbinafine was used as positive control for fungal strains MIC (μg/mL): CA = 6.25; CT = 6.25; CP = 0.125; AB = 25.0. The extract with the most promising antimicrobial activity is in bold.

**Table 3:** Antioxidant activity of the extracts of endophytic fungi from *Arrabidaea chica*. Antioxidant activity (AA) obtained using the DPPH method, the efficient concentration for the sequestration of 50% of the DPPH• free radicals (EC_50_), and the ferric reducing antioxidant power (FRAP).

Endophytic Fungi Code	AA *(%)	EC_50_ (μg/mL)	FRAP * (µmol TE/g)	Endophytic Fungi Code	AA * (%)	EC_50_ (μg/mL)	FRAP * (µmol TE/g)
CF1-3	98.61 ^a^	5490	110.6 ^B^	CF2-11	100.0 ^a^	1080	128.5 ^B^
CF1-4	100.0 ^a^	6870	77.4 ^C^	CF2-12	86.32 ^b^	6450	93.6 ^C^
CF1-7	92.38 ^b^	1250	174.4 ^A^	**CF2-13**	**92.47 ^b^**	**360**	71.9 ^C^
CF1-9	90.74 ^b^	5530	143.2 ^B^	CF2-14	90.22 ^b^	1620	167.9 ^A^
CF1-12	90.74 ^b^	5270	95.2 ^C^	**CF2-16**	94.81 ^b^	1170	**218.6 ^A^**
**CF1-13**	91.43 ^b^	6480	**214.0 ^A^**	CF2-17	83.03 ^c^	6820	48.0 ^C^
CF1-15	94.20 ^b^	2710	171.0 ^A^	CF2-18	70.74 ^d^	7540	46.5 ^C^
CF1-16	92.81 ^b^	6190	72.4 ^C^	CF2-20	82.68 ^c^	1660	192.5 ^A^
CF1-18	88.14 ^b^	2840	178.6 ^A^	CG2-2	95.06 ^b^	3200	103.3 ^B^
CF1-19	82.77 ^c^	6590	47.5 ^C^	CG2-4	91.95 ^b^	1520	151.3 ^A^
**CF1-20**	**100.0 ^a^**	**990**	121.9 ^B^	CG2-5	89.87 ^b^	2720	51.0 ^C^
CF1-23	94.20 ^b^	5730	154.0 ^B^	CG2-7	95.76 ^b^	4710	59.7 ^C^
CF1-24	89.70 ^b^	3420	39.1 ^C^	**CG2-10**	**98.01 ^a^**	**740**	**191.2 ^A^**
CF1-25	93.59 ^b^	1060	188.4 ^A^	CG2-12	95.75 ^b^	4750	169.6 ^A^
CF1-26	81.13 ^c^	6720	177.3 ^A^	CG2-16	89.78 ^b^	3250	86.1 ^C^
CF1-27	95.93 ^b^	1460	125.2 ^B^	CF3-1	95.06 ^b^	2390	133.4 ^B^
CF1-28	93.77 ^b^	5020	181.7 ^A^	CF3-4	90.04 ^b^	6280	197.3 ^A^
CF1-29	93.85 ^b^	2260	109.1 ^B^	**CF3-5**	**86.23 ^c^**	**940**	**161.9 ^B^**
CF1-30	88.83 ^b^	3060	197.5 ^A^	CF3-9	100.0 ^a^	2480	142.3 ^B^
CF1-31	100.0 ^a^	1410	55.9 ^C^	CF3-11	80.26 ^b^	8310	53.2 ^C^
CF1-36	88.23 ^b^	2550	82.6 ^C^	CF3-13	98.35 ^a^	65,050	53.5 ^C^
**CF1-37**	**93.68 ^b^**	**680**	**171.1 ^A^**	CF3-14	95.76 ^b^	5400	152.8 ^B^
CG1-1	90.56 ^b^	2920	131.7 ^B^	CF3-16	91.52 ^b^	6100	158.2 ^B^
CG1-2	90.39 ^b^	3560	148.6 ^B^	CF3-17	76.97 ^c^	7610	56.9 ^C^
CG1-4	89.78 ^b^	1060	199.0 ^A^	CF3-18	100.0 ^a^	5650	175.8 ^A^
CG1-5	92.38 ^b^	5770	166.0 ^A^	CF3-20	90.30 ^b^	6250	148.5 ^B^
**CG1-7**	**100.0 ^a^**	**11**	109.5 ^B^	CF3-21	85.89 ^c^	6820	139.7 ^B^
CG1-10	89.44 ^b^	6420	44.7 ^C^	CF3-26	88.48 ^b^	3080	172.7 ^A^
CG1-11	87.97 ^b^	6660	128.2 ^B^	CG3-3	80.00 ^c^	5200	83.1 ^C^
CG1-12	88.92 ^b^	3700	89.5 ^C^	CG3-4	90.04 ^b^	4830	114.4 ^B^
CG1-14	81.99 ^c^	2880	154.8 ^B^	**CG3-7**	**79.91 ^c^**	**500**	**167.0 ^A^**
CF2-2	75.06 ^c^	6170	106.7 ^B^	CG3-8	89.27 ^b^	7300	79.9 ^C^
CF2-6	80.43 ^c^	5850	82.0 ^C^	CG3-13	77.40 ^c^	7610	169.3 ^A^
CF2-7	78.61 ^c^	4100	59.2 ^C^	CG3-18	97.75 ^a^	5670	158.5 ^B^
CF2-9	94.98 ^b^	5630	78.9 ^C^	CG3-19	91.17 ^b^	6210	96.2 ^C^
**Quercetin**	**98.00 ^a^**	**8**	NT	**Ascorbic acid**	NT	NT	163.1 ^A^

* Assays carried out with the fungal extracts at a concentration of 10 mg/mL. Quercetin and ascorbic acid were tested at 40 μg/mL. NT = not tested. Results are expressed as means of the experiments in triplicate. Means that do not share a letter are significantly different (*p* < 0.05) according to the Bonferroni test. The extracts with the most promising antioxidant action are in bold.

**Table 4:** GenBank accession numbers for the fungal isolates from *Arrabidaea chica* producing bioactive metabolites. Newly deposited sequences are shown in bold.

			GenBank Accession Number		
Isolate	Species	Source	ITS	Βtub	CaM
CG1-7	*Colletotrichum* sp.	*A. chica*	**OQ390099**	**OQ412637**	**OQ412636**
CF2-13	*Botryosphaeria mamane*	*A. chica*	**OQ696843**	**OQ703591**	*-

* No resolution. ITS = internal transcribed spacer region. βtub = β-tubulin. CaM = calmodulin.

## Data Availability

No new data were created or analyzed in this study. Data sharing is not applicable to this article.
